# Effect of Promotional Initiatives on Visits to a Dedicated Website for Physical Activity and Non-Communicable Disease in Luxembourg: An Event Study

**DOI:** 10.3389/fpubh.2017.00114

**Published:** 2017-05-29

**Authors:** Alexis Lion, Jane S. Thornton, Michel Vaillant, Juliette Pertuy, Eric Besenius, Cyrille Hardy, Charles Delagardelle, Romain Seil, Axel Urhausen, Daniel Theisen

**Affiliations:** ^1^Sports Medicine Research Laboratory, Luxembourg Institute of Health, Luxembourg City, Luxembourg; ^2^Western Centre for Public Health and Family Medicine, University of Western Ontario, London, ON, Canada; ^3^Competence Center in Methodology and Statistics, Luxembourg Institute of Health, Luxembourg City, Luxembourg; ^4^Communication, Luxembourg Institute of Health, Luxembourg City, Luxembourg; ^5^Service de Cardiologie, Centre Hospitalier de Luxembourg, Luxembourg City, Luxembourg; ^6^Service de Chirurgie Orthopédique, Centre Hospitalier de Luxembourg, Luxembourg City, Luxembourg; ^7^Clinique du Sport, Centre Hospitalier de Luxembourg, Luxembourg City, Luxembourg; ^8^LUNEX University, Differdange, Luxembourg

**Keywords:** event study, Internet, health promotion, non-communicable diseases, sport

## Abstract

The Sport-Santé project and its website (www.sport-sante.lu) promote physical activity for individuals with non-communicable diseases (NCDs) in Luxembourg. Our purpose was to perform an event study analysis to evaluate the effects of communication and promotional initiatives on the number of visits to the Sport-Santé website. Between September 2015 and May 2016, the Sport-Santé website was promoted during different initiatives, including participation in health-related events or publication of articles in local journals. The daily number of visits to www.sport-sante.lu website (i.e., our outcome) was recorded using Google Analytics and compared to a counterfactual collected with its benchmarking tool. The counterfactual was defined as the daily number of visits to websites in the same field. A model was created to evaluate the relationship between the number of visits to www.sport-sante.lu website and the number of visits to similar websites during a control period with no promotional initiatives (from July 2015 to September 2015). The effect of promotional initiatives was subsequently tested, by comparing the actual number of visits to our website (up to 2 days after each event) with the theoretical number of visits predicted by the model. Twenty-two initiatives were identified, of which 11 were participations at major health-related events and 11 publications of popular science articles. Of these 22 initiatives, the event study identified 2 popular science articles and 1 interactive workshop that significantly increased the daily number of visits to the www.sport-sante.lu website. One of the two articles was published on the day before the workshop was held, which did not allow us to distinguish its specific impact. The second article was published in the main national newspaper. This is the first time to our knowledge that an event study analysis has been used to evaluate the impact of promotional initiatives on the number of visits to a dedicated website for physical activity and NCDs. Our results indicate that some initiatives can aid in the number of visits, but in general their impact is limited. To observe an increased rate of participation in physical activity, additional promotional and evaluative strategies should be explored.

## Introduction

Globally, physical inactivity causes more than five million deaths each year (1). It is considered by the World Health Organization (WHO) as one of the leading risk factors for non-communicable diseases (NCDs) such as cardiovascular disease, stroke, obesity, or cancer. The association between physical activity and cardiovascular disease has been described extensively, whereby mortality risk is inversely related to the level of physical activity (2). Moreover, a recent study showed that physical activity was associated with lower risks of 10 types of cancer, independently of other risk factors such as obesity or smoking history (3). Physical activity is not only effective in the primary prevention of major NCDs but also confers benefits during and after treatment (4). It improves cardiac performance and muscle strength in patients with NCDs and reduces fatigue, anxiety, and depression (4). In addition, balance, mobility, and coordination are enhanced in physically active patients (4). Therefore, physical activity could be considered a therapy (5–7). For some NCDs, such as cardiovascular disease or breast cancer, physical activity can reduce the recurrence of the disease following remission (8, 9).

In light of this evidence, various forms of physical activity have been suggested for people with cardiovascular diseases for several decades. In the Grand-Duchy of Luxembourg (a European country with 0.5 million inhabitants), the first non-governmental organization offering physical activity for people with cardiovascular diseases was founded in 1984 (10). A number of other organizations followed suit and began offering physical activity sessions to obese patients, and patients with cancer, rare diseases, or neurologic and orthopedic conditions (11). The participation rate in these sessions, however, has been relatively low compared to the number of potential participants (11). The Sport-Santé project (“*Santé*” being the French word for health) was launched in early 2015 to help these organizations better promote the physical activity training they offer in the Grand-Duchy of Luxembourg.

**Figure 1:**
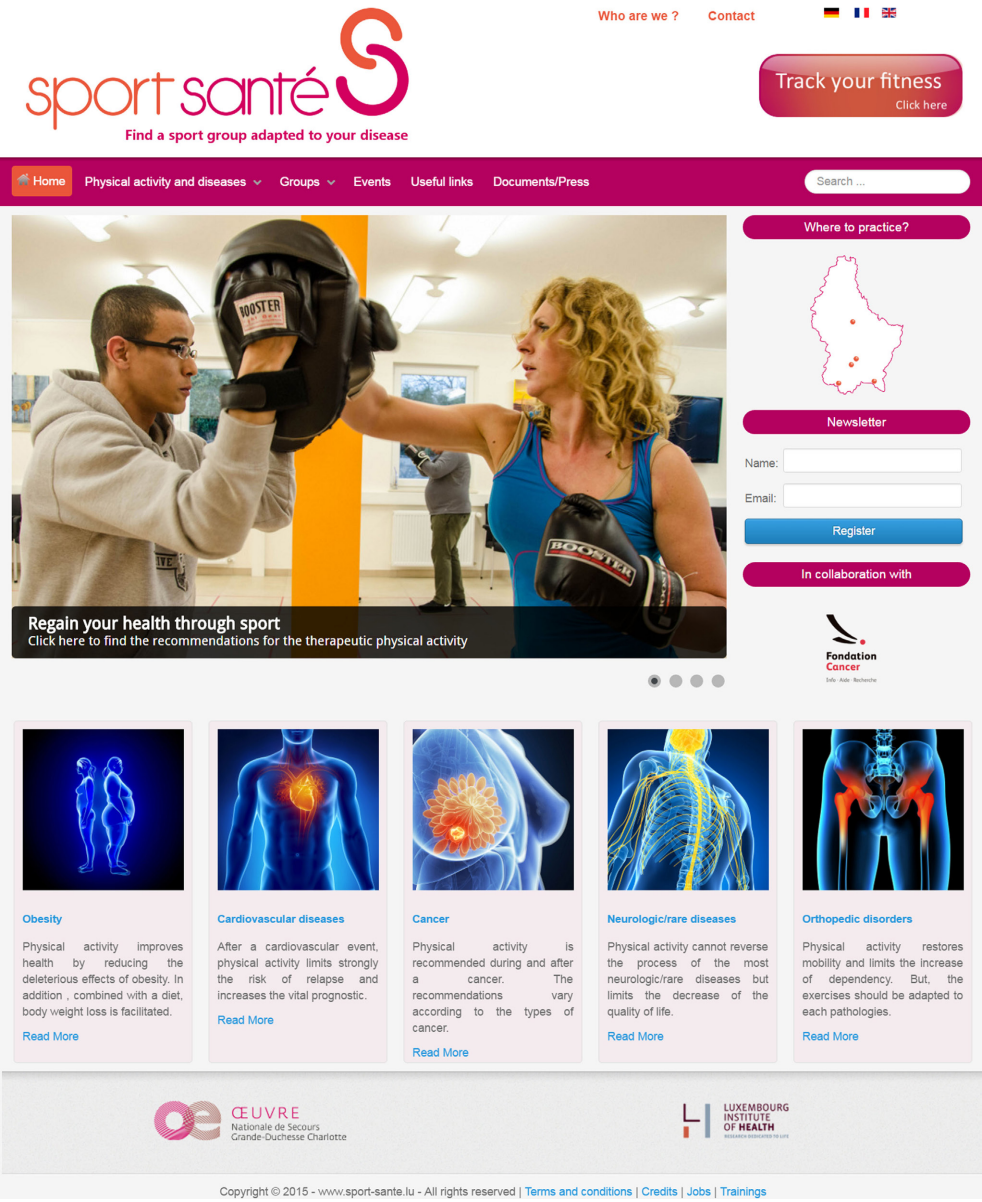
**Screenshot of the Sport-Santé homepage (www.sport-sante.lu)**. While the local offer of therapeutic physical activity is available within the “Groups” section and located on the map, the theoretical factsheets can be found in the “Physical activity and diseases” section. The homepage contains also links to the events and the Sport-Santé documents. With the kind permission of the Oeuvre Nationale de Secours Grande-Duchesse Charlotte.

The first aim of this project was to compile and list the various opportunities available for physical activity and display them on a dedicated website (www.sport-sante.lu—Figure 1), which was inspired by similar websites of other countries: www.fyss.se in Sweden, www.sport-sante.fr in France, www.sportprogesundheit.de in Germany, www.paprica.ch in Switzerland, or www.exerciseismedicine.com.au in Australia. The Sport-Santé website provides accurate and current information regarding the local offer of therapeutic physical activity (group factsheets: e.g., “Association Luxembourgeoise des Groupes Sportifs pour Cardiaques,”^1^ “Fondation Cancer,”^2^ “Parkinson Luxembourg”^3^) being displayed on a map. Moreover, it contains theoretical factsheets explaining the rationale to practice physical activity according to different diseases (e.g., “Coronary heart disease and physical activity,”^4^ “Breast cancer and physical activity,”^5^ “Parkinson’s disease and physical activity,”^6^). The website also provides a list of past and future events,^7^ contacts, and a page containing useful documents and press.^8^ After the launch of the website in April 2015, several promotional initiatives were undertaken to promote the project and its website. Popular science publications for the local press were written concerning the health benefits of practicing physical activity during or after treatments of NCDs (e.g., “Sport and Cancer: for who, why, how?”^9^). The readers were invited to visit the Sport-Santé website for more information. In addition, participation at health- and/or sport-related public events to promote physical activity for people with NCDs (e.g., World Heart Day organized in the lobby of a hospital or World Family Doctor Day organized in the center of Luxembourg City) were also included in the communication strategy of the Sport-Santé project. At these events, Sport-Santé was promoted *via* booths with promotional materials such as banners, posters, and flyers encouraging people to visit the website. During discussion with visitors at the Sport-Santé booths, the exhibitors invited them to visit the website for more theoretical and practical details.

However, the number of participants involved in physical activity opportunities offered by the different Luxembourgish organizations had not increased 1 year after the launch of the website (12). The conclusion would seem to be that the goal of the Sport-Santé project has not yet been realized, despite the previously mentioned promotional strategies. One reason for this could be a patients’ lack of awareness of the projects, raising the question of the effectiveness of our promotional initiatives to reach these patients. We speculated that an increase in website visits would represent a greater awareness of the Sport-Santé project which then could lead to an increase in participation in the dedicated groups and ultimately a higher level of physical activity. Evaluating the impact of promotional initiatives is thus important to optimize communication. In this study, we used an event study analysis to evaluate the impact of our promotional initiatives on the number of visits to our website promoting physical activity for secondary and tertiary prevention of NCDs.

## Materials and Methods

### Event Study Analysis

#### Event Study Methodology

Event study is a statistical method used to evaluate the impact of an event on an outcome. This method is a comparison between what actually happened and what would have happened in the absence of the event. It has been used mainly in economics (13), but is also used in other research areas, such as accounting and finance, management, marketing, information technology, law, and political science. Completing an event study requires different steps such as data collection with respect to the outcome and its counterfactual (i.e., the reference value), identification of the period of interest and the events, and, finally, statistical analysis (14).

#### Data Collection of the Number of Visits to www.sport-sante.lu

To measure how users interact with our website, a JavaScript tracking snippet sending browsing data to Google Analytics was added to each website page during the development phase of www.sport-sante.lu. Google Analytics was used to collect website usage information from April 22, 2015 (website launch) to May 26, 2016 included. Before exporting data for statistical analysis, a country/region filter was applied to the data. The daily numbers of visits to the website were thus searched for the Grand-Duchy of Luxembourg as well as the surrounding area: Belgium, Lorraine (France), Rhineland-Palatinate (Germany), and Saarland (Germany). The number of visits per day was considered a marker of the popularity of the website.

#### Data Collection of the Counterfactual

The counterfactual is the reference value and is mandatory for statistical analysis. Therefore, in the current study, it was defined as the daily number of visits to websites in the same field and geographic region as the Sport-Santé website. This number was collected through the Google Analytics Benchmark tool over the same time period. Benchmarking provided a website visit mean value from aggregate data of other websites sharing their data. A benchmark selection was therefore necessary to obtain a counterfactual, which could be comparable to the number of visits to the Sport-Santé website. The selection was based on three filters: industry, country/region, and number of daily visits. For the first benchmark selection, the three filters were “all sciences” (i.e., the type of industry), “Luxembourg” (i.e., country/region), and “0–99” (i.e., the range of number of daily visits), respectively. For the second benchmark selection, the three filters were “all sports,” “Luxembourg,” and “0–99,” respectively. The data (daily number of visits) of the two benchmark selections were exported and averaged.

#### Estimation

**Figure 2:**
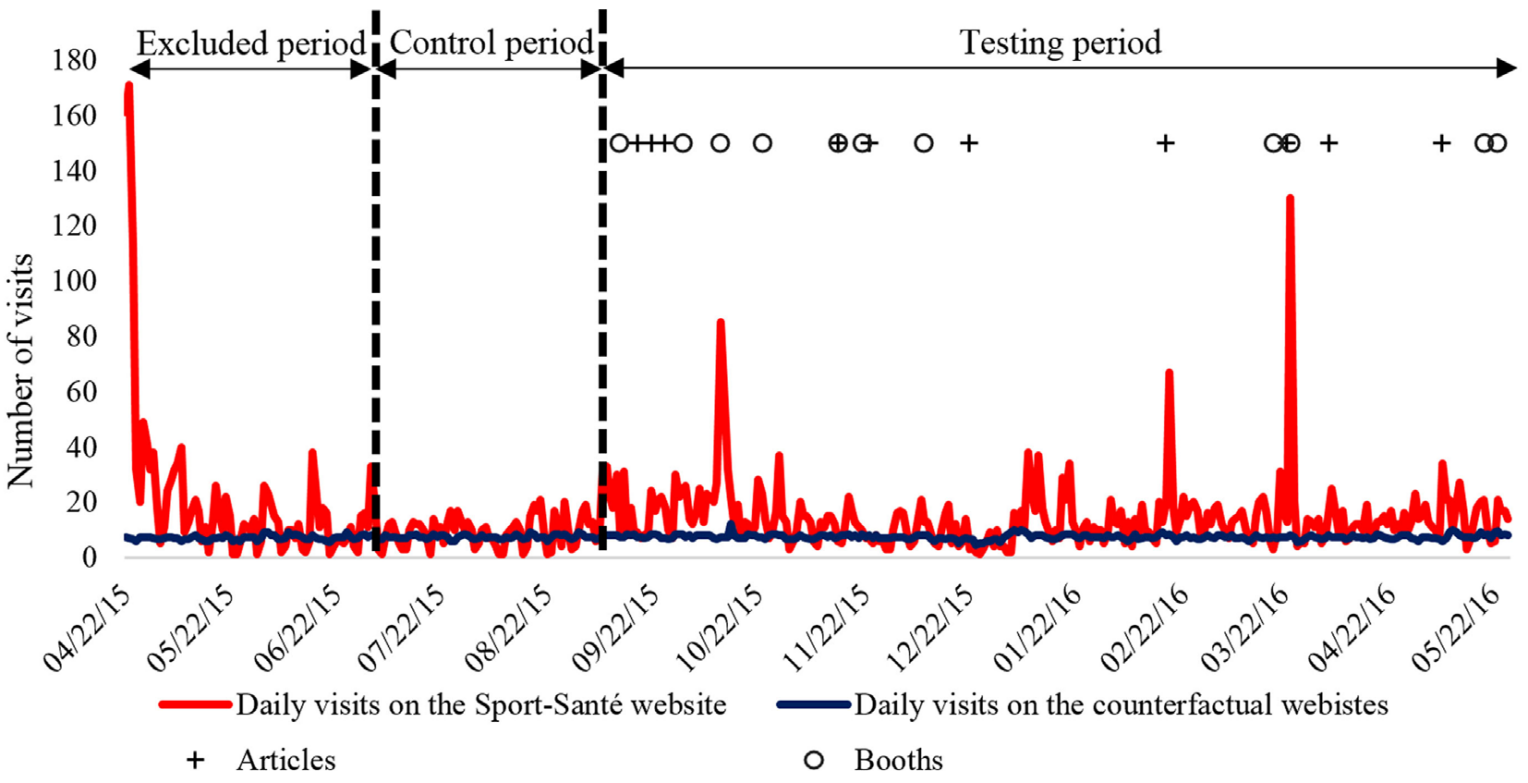
**Number of the daily visits on the Sport-Santé website (dark red line) and the counterfactual websites (dark blue line) from April 22, 2015 to May 25, 2016**. The period from the launch of the Sport-Santé website (April 22, 2015) to July 5, 2015 was excluded from the analysis. The period from July 5, 2015 to September 5, 2015 was considered as a control period. The effect of the advertising on the number of visits to the website was tested from September 5, 2015 to May 25, 2016 (testing period). The articles and the booths which promote Sport-Santé are identified by crosses and circles, respectively.

The aim of our event study was to analyze whether an initiative induced an abnormal return of the daily number of visits on www.sport-sante.lu. The abnormal return is the difference between the actual return (*r*) of the outcome and the expected return ([E]*r*) of the counterfactual. Logarithmic returns were used both for the returns of the outcome and its counterfactual and calculated as follows: *r* = ln(*V*_f_/*V*_i_), where *V*_f_ and *V*_i_ were the final and initial daily number of visits. A control period, which was defined prior to the studied events and did not include any promotional initiative, was identified from July 5, 2015 to September 5, 2015. The period from April 22, 2015 (website launch) to July 5, 2015 was excluded in order to avoid the short-term effects of the website launch on the daily number of visits (Figure 2). The control period was used to derive the typical relationship between the returns of the outcome (i.e., daily number of visits to www.sport-sante.lu) and its counterfactual (daily number of visits from the Google Analytics Benchmark tool) through regression analysis. Coefficients (intercept and slope) from this regression analysis were incorporated in combination with the logarithmic return of the counterfactual (*r*_c_) to calculate the expected return ([E]*r*) as follows: [E]*r* = intercept + slope × *r*_c_. The daily abnormal returns (AR) were calculated as follows: AR = *r*_a_ − [E]*r*, where *r*_a_ is the actual return of the outcome (daily number of visits on www.sport-sante.lu). The significance of the AR was tested as follow: *t* = AR/SE, where SE is the standard error of the control period determined in the regression analysis (SE was calculated as follows: SE = SD/√*n*, where SD is the standard deviation and *n* is the number of observations during the control period). A *p*-value of 0.05 was considered significant. For each initiative identified, an event period of 3 days (including the event day) was set to evaluate the potential short-term delayed effect of the communication. In addition, a cumulative abnormal return (CAR) was calculated based on the daily AR to ascertain the magnitude of the AR overt the entire event window.

#### Sport-Santé Initiatives

All initiatives promoting physical activity among people with NCDs in Luxembourg in the setting of the Sport-Santé project were considered from September 2015 to May 2016. Initiatives included publication of popular science articles, presentations at information booths and workshops, and participation at different major health-related events in Luxembourg. The Sport-Santé website (www.sport-sante.lu) was recommended at each initiative. The organizers or the editors, the attendance or the reprints, and the targeted audience were retrospectively searched for each initiative. The event study analysis was applied for each initiative.

### Page Views on www.sport-sante.lu

In addition to the event study analysis, the total number of page views and the number of page views per section (homepage, groups’ factsheets, theoretical factsheets, events, contacts, press/links, map, search, others) were investigated using Google Analytics for the control and testing periods.

## Results

For the counterfactual, the daily number of visits to 375 websites for the first benchmark selection and 396 websites for the second benchmark selection was averaged. Data from the Sport-Santé website and the counterfactual are presented in Figure 2. The coefficients of the regression analysis evaluating the relationship between the returns of outcome and its counterfactual during the control period are as follows: slope = 4.58, intercept = 0.028, *r*-squared = 0.30, and SE = 0.81.

From September 5, 2015 to May 25, 2016, 22 initiatives were identified. Sport-Santé was promoted *via* a booth at 11 events across the country (Table 1). These events were mainly organized by health associations (cancer patients, cardiac patients, physiotherapists, general practitioners) and targeted a broad audience (general population, policymakers, patients, and health-care professionals). At all of these events, Sport-Santé was highlighted along with the other participating organizations. Attendance ranged from 50 to an estimated 13,000 people. In addition, 11 popular science articles were published in the local newspapers, local healthcare press, and journals of different health associations (for cancer patients, cardiac patients, patients with Parkinson’s disease, general practitioners) to promote Sport-Santé (Table 2). In the articles, the project Sport-Santé was described and the Internet address was provided. All the articles contained theoretical information regarding the benefits of physical activity in the management of NCDs, which were often highlighted with testimonials. The targeted readers were mainly patients and healthcare professionals. The number of reprints ranged from 1,600 to 88,000.

**Table 1:** **List of the booths aiming to promote Sport-Santé in Luxembourg**.

Event’s name	Date	Organizers	Attendance	Targeted audience
*Salon Top Sport*	September 12, 2015	Ministry for Sport	4,000	Policymakers, family, sport federation
*World Heart Day*	September 30, 2015	Hospital, research institute, sport association for cardiac patients	Unknown	Patients and relatives, health-care professionals
*Marathon Indoor Cycling*	October 11, 2015	Association for renal failure patients	1,024	Policymakers, renal failure patients, sympathizers, health-concerned persons
*City Concorde*	October 23, 2015	Mall, association for breast cancer patients	13,000^a^	Mall clients
*30 ans ALGSC*	November 14, 2015	Hospital, sport association for cardiac patients	200	Policymakers, cardiologists, patients and relatives
*Journée Kiné ALK*	November 21, 2015	Association of physiotherapists	50	Physiotherapists
*Assemblée Générale AMMD*	December 09, 2015	Association of general practitioners	91	General practitioners
*Relais pour la Vie*	March 19, 2016	Association for cancer patients	12,565	Policymakers, cancer patients and relatives, sympathizers
*Health Virus Day*	March 24, 2016	School	400	Nurse and health-care students
*World Family Doctor Day*	May 19, 2016	Association of general practitioners	100	General population, general practitioners
*European Obesity Day*	May 23, 2016	Hospital	Unknown	Patients and relatives, health-care professionals

*^a^Estimation based on an article published in a local and economical newspaper (Clarinval F. 50 millions d’investissements pour City Concorde. Paperjam, March 2015)*.

**Table 2:** **List of the published popular science articles aiming to promote Sport-Santé in Luxembourg**.

Journal name	Date	Editors	Reprints	Targeted audience
*FHL Info* (no. 31)	September 17, 2015	Local health-care press	2,000	Hospital waiting rooms
*Semper* (no. 72)	September 21, 2015	Local health-care press	2,600	General practitioners, scientists
*Info Cancer* (no. 82)	September 25, 2015	Association for cancer patients	88,000	Cancer patients, health-concerned persons
*30 ans ALGSC*	November 14, 2015	Sport association for cardiac patients	3,500	Policymakers, cardiologists, patients, and relatives
*Corps Médical* (no. 10)	November 23, 2015	Association of general practitioners	1,538	General practitioners
*Flambeau* (no. 81)	December 22, 2015	Local Olympic committee	7,000	Sport-concerned persons
*Semper* (no. 77)	February 17, 2016	Local health-care press	2,600	General practitioners, scientists
*Letz Be Healthy* (no. 4)	February 17, 2016	Local health-care press	20,000	Clients of the pharmacies
*Letz Be Healthy* (no. 5)	March 23, 2016	Local health-care press	20,000	Clients of the pharmacies
*Bulletin officiel Parkinson Luxembourg* (no. 47)	April 04, 2016	Association for patients with Parkinson Disease	1,600	Patients and relatives
*Luxemburger Wort*	May 07, 2016	Local written press	69,700	General population

**Figure 3:**
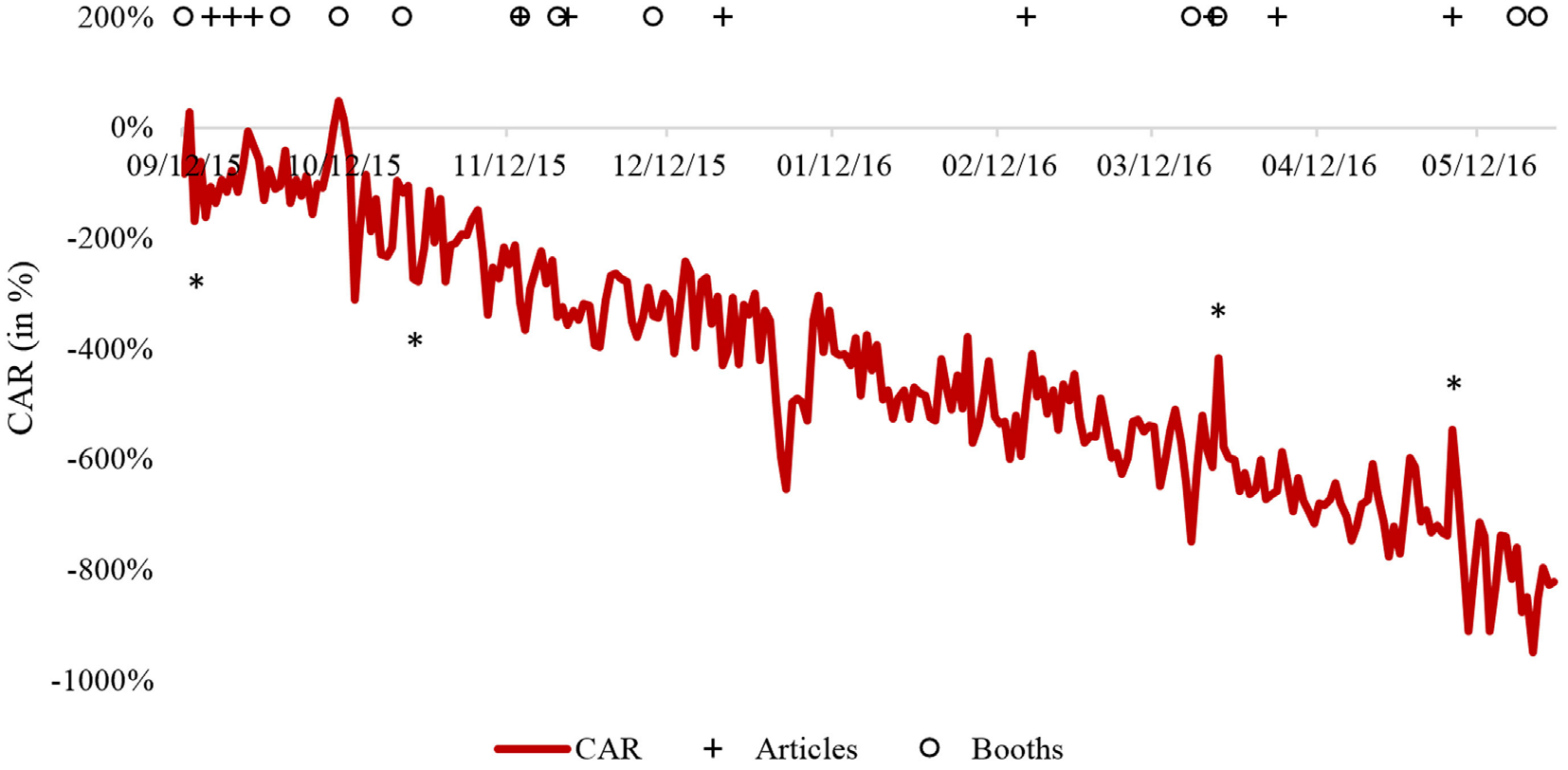
**Cumulative abnormal returns (*CAR*) for the testing period**. The articles and the booths to promote Sport-Santé are identified by crosses and circles, respectively. *Statistically significant initiatives revealed by the event study (*p* < 0.05).

The event study revealed that only three initiatives [*Health Virus Day, Letz Be Healthy* (no. 5), and *Luxemburger Wort*] had AR, which were statistically significantly higher than the [E]*r* (Tables 3 and 4). For the *Health Virus Day* and the first day after the publication of a popular science article in the magazine *Letz Be Healthy* (no. 5) (March 24, 2016), the AR was 198% while the [E]*r* was 33% (*p* = 0.020). For the popular science article published in the *Luxemburger Wort* on May 7, 2016, the AR was 192% while the [E]*r* was −34% (*p* = 0.024). However, all these elevated AR were immediately followed by a (non-significant) negative AR ranging from −160 to −122%, highlighting that the effect is not maintained. Indeed, the significant positive effect of these three initiatives was not able to protect against the decrease of the *CAR* during the entire event window (Figure 3). In addition, the event study analysis revealed that two initiatives (*Salon Top Sport, City Concorde*) had *AR*, which were statistically significantly lower than the [E]*r* for the second day following the event (Table 3).

**Table 3:** **Expected return ([E]*r*) and abnormal return (AR) for each booth and the 2 days following each event**.

Event	Date (mm/dd/yyyy)	Day	[E]*r* (in %)	AR (in %)	*t*-Value	*p*-Value
*Salon Top Sport*	09/12/2015	Day 0	−27	−83	−1.03	0.23
	09/13/2015	Day 1	3	110	1.36	0.15
	09/14/2015	Day 2	60	−196	−2.42	0.021

*World Heart Day*	09/30/2015	Day 0	3	6	0.07	0.39
	10/01/2015	Day 1	−55	63	0.77	0.29
	10/02/2015	Day 2	33	−94	−1.17	0.20

*Marathon Indoor Cycling*	10/11/2015	Day 0	69	46	0.57	0.34
	10/12/2015	Day 1	3	−33	−0.41	0.37
	10/13/2015	Day 2	3	−71	−0.87	0.27

*City Concorde*	10/23/2015	Day 0	3	−23	−0.28	0.38
	10/24/2015	Day 1	−63	13	0.16	0.39
	10/25/2015	Day 2	98	−167	−2.07	0.047

*30 ans ALGSC*	11/14/2015	Day 0	35	−104	−1.28	0.17
	11/15/2015	Day 1	33	−51	−0.63	0.33
	11/16/2015	Day 2	3	76	0.94	0.26

*Journée Kiné ALK*	11/21/2015	Day 0	92	−102	−1.26	0.18
	11/22/2015	Day 1	−55	19	0.23	0.38
	11/23/2015	Day 2	33	−33	−0.40	0.36

*Assemblée Générale AMMD*	12/09/2015	Day 0	3	−51	−0.63	0.33
	12/10/2015	Day 1	3	−3	−0.04	0.40
	12/11/2015	Day 2	−92	44	0.54	0.34

*Relais Pour la Vie*	03/19/2016	Day 0	35	−104	−1.28	0.17
	03/20/2016	Day 1	−29	139	1.71	0.09
	03/21/2016	Day 2	35	89	1.10	0.21

*Health Virus Day*	03/24/2016	Day 0	33	198	2.45	0.020
	03/25/2016	Day 1	−27	−160	−1.98	0.06
	03/26/2016	Day 2	−139	−22	−0.27	0.38

*World Family Doctor Day*	05/19/2016	Day 0	−51	56	0.69	0.31
	05/20/2016	Day 1	31	−115	−1.43	0.14
	05/21/2016	Day 2	−86	27	0.34	0.37

*European Obesity Day*	05/23/2016	Day 0	28	98	1.21	0.19
	05/24/2016	Day 1	−76	55	0.68	0.32
	05/25/2016	Day 2	31	-31	-0.38	0.37

**Table 4:** **Expected return ([E]*r*), and abnormal return (AR) for each popular science article and the 2 days following each publication**.

Publication	Date (mm/dd/yyyy)	Day	[E] *r* (in %)	AR (in %)	*t*-Value	*p*-Value
*FHL Info* (no. 31)	09/17/2015	Day 0	−55	55	0.67	0.23
	09/18/2015	Day 1	3	−28	−0.35	0.37
	09/19/2015	Day 2	−29	42	0.52	0.35

*Semper* (no. 72)	09/21/2015	Day 0	60	38	0.47	0.36
	09/22/2015	Day 1	3	−37	−0.46	0.37
	09/23/2015	Day 2	−25	46	0.57	0.34

*Info Cancer* (no. 82)	09/25/2015	Day 0	3	−23	−0.28	0.38
	09/26/2015	Day 1	−31	−28	−0.34	0.38
	09/27/2015	Day 2	37	−73	−0.90	0.27

*30 ans ALGSC*	11/14/2015	Day 0	35	−104	−1.28	0.17
	11/15/2015	Day 1	33	−51	−0.63	0.33
	11/16/2015	Day 2	3	76	0.94	0.26

*Corps Médical* (no. 10)	11/23/2015	Day 0	33	−33	−0.40	0.37
	11/24/2015	Day 1	−58	25	0.31	0.38
	11/25/2015	Day 2	64	−17	−0.21	0.39

*Flambeau* (no. 81)	12/22/2015	Day 0	−31	−123	−1.52	0.12
	12/23/2015	Day 1	3	26	0.32	0.38
	12/24/2015	Day 2	−166	97	1.19	0.19

*Letz Be Healthy* (no. 4) and *Semper* (no. 77)	02/17/2016	Day 0	−51	94	1.17	0.20
	02/17/2016	Day 1	31	90	1.12	0.21
	02/17/2016	Day 2	−55	−77	−0.95	0.25

*Letz Be Healthy* (no. 5)	03/23/2016	Day 0	3	−30	−0.37	0.37
	03/24/2016	Day 1	33	198	2.45	0.020
	03/25/2016	Day 2	−27	−160	−1.98	0.06

*Bulletin Officiel Parkinson Luxembourg* (no. 47)	04/04/2016	Day 0	64	5	0.06	0.39
	04/05/2016	Day 1	−27	71	0.88	0.27
	04/06/2016	Day 2	3	−48	−0.59	0.33

*Luxemburger Wort*	05/07/2016	Day 0	−34	192	2.37	0.024
	05/08/2016	Day 1	74	−122	−1.51	0.13
	05/09/2016	Day 2	118	−118	−1.46	0.14

**Figure 4:**
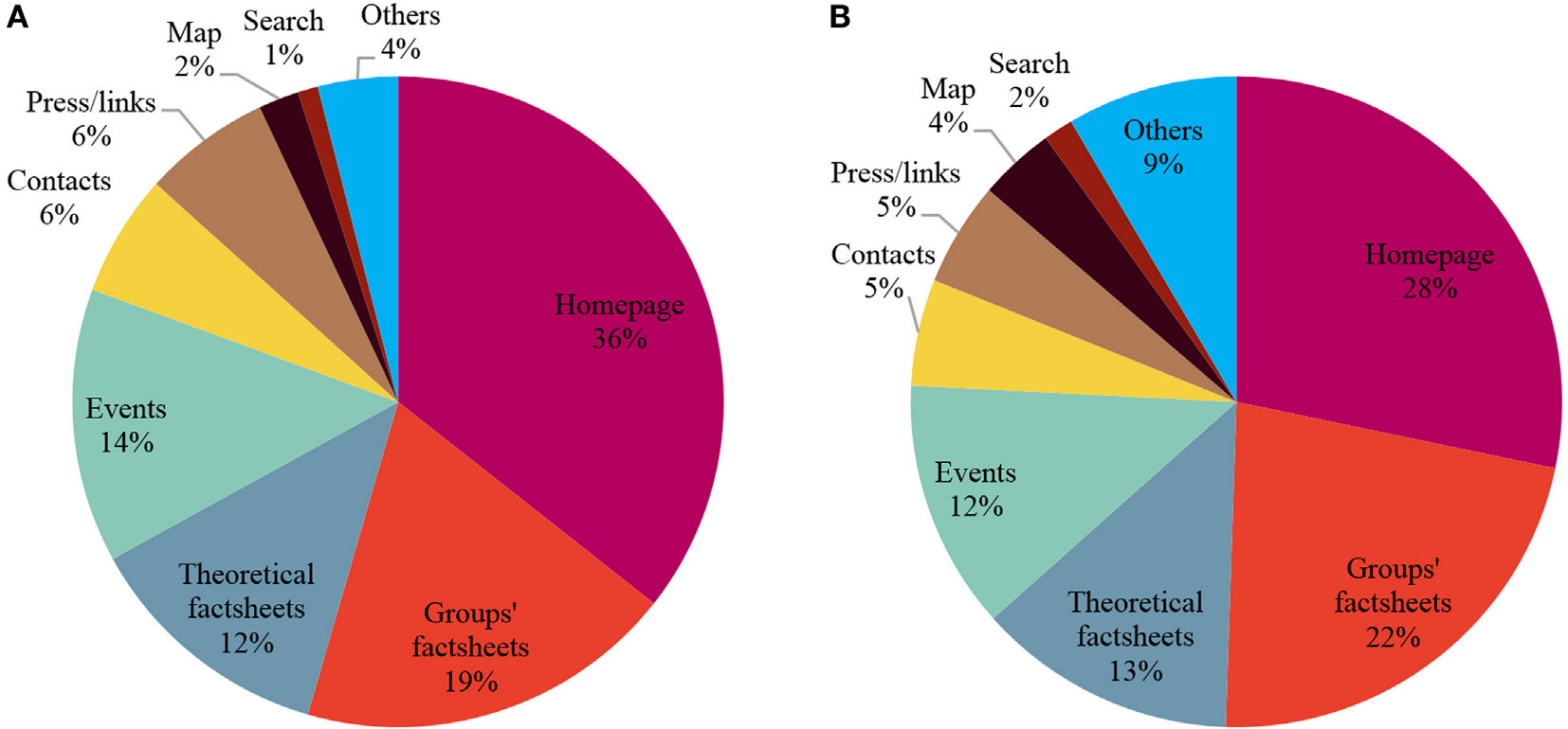
**Pie charts of the percentages of the page views per section on www.sport-sante.lu during the control [(A), total number of viewed pages: 1,990] and the testing [(B), total number of viewed pages: 12,087] periods**.

During the control period, 1,990 pages were visited. The three most viewed sections (Figure 4A) were the homepage (36%), the groups’ factsheets (19%), and the theoretical factsheets (12%). During the testing period, 12,087 pages were visited. The three most viewed sections (Figure 4B) were again the homepage (28%), the groups’ factsheets (22%), and the theoretical factsheets (13%).

## Discussion

This study showed that only three initiatives promoting physical activity for patients with NCDs in the Grand-Duchy of Luxembourg had a significant positive effect on the daily number of visits to www.sport-sante.lu.

The promotion of physical activity for patients with NCDs is growing worldwide and is targeted in different ways. The role of technology to promote physical activity has indeed been increasing consistently within the last two decades. Internet and mobile interventions are reported to significantly improve the level of physical activity in adults who are healthy or at risk of diabetes or hypertension (15–17). Most of these interventions include regular emails, text messages, phone counseling, apps, and/or websites. Promising results are observed also for patients with NCDs. Indeed, Internet-based interventions are effective at increasing physical activity in patients with cardiovascular disease or cancer (especially the youngest patients) (18, 19). In addition to research in e-health interventions, several websites, including practical and theoretical information regarding the primary, secondary, and tertiary prevention of NCDs, have been developed in different European countries: www.fyss.se in Sweden, www.sport-sante.fr in France, https://gpcpd.walesdeanery.org/index.php/welcome-to-motivate-2-move in Wales, www.sportprogesundheit.de in Germany, www.paprica.ch in Switzerland, and www.exercise-works.org in the UK. However, the effectiveness of such websites to increase the level of physical activity of patients with NCDs without targeted interventions using emails or text messages is still unknown. Hence, it was deemed urgent to develop, promote, and evaluate such a website for the Grand-Duchy of Luxembourg as well.

Evaluation of the promotional initiatives was done to help determine those that work and those that do not. Three effective initiatives were identified in our event study: two popular science publications (*Letz Be Healthy* no. 5 and *Luxemburger Wort*) and one interactive workshop (*Health Virus Day*). The article concerning Sport-Santé in the *Letz Be Healthy*, however, was published the day before the *Health Virus Day* workshop. Therefore, the overlapping of these two events means it cannot be determined if the effect on the number of visits to the Sport-Santé website was due more to one or the other. *Letz Be Healthy* is a new magazine distributed monthly to pharmacies in the Grand-Duchy of Luxembourg. This free magazine has a run of 20,000 printed copies each month. *Health Virus Day* is an event organized by a healthcare school (*Lycée Technique pour Professions de Santé*) to sensitize almost 400 nursing students to different topics concerning health and physical activity. A Sport-Santé workshop was held and the participating students were asked to search for information on www.sport-sante.lu to complete a quiz. Even if nursing students were not the primary target of the Sport-Santé project, this workshop contributed to a consistent increase in the number of visits to the website. The third significant initiative was the publication of an article in the *Luxemburg Wort*, which is the most-read newspaper in Luxembourg with a circulation of almost 70,000 (175,200 daily readers for print and e-paper). The 19 remaining initiatives (9 articles and 10 public events) did not have a significant positive effect on the daily number of visits to the website. The audience of these nine articles was more limited. The 10 public events did not include actions specifically asking people to access the website. The content of the booth and the attitude of the exhibitors could also have played a role in the effectiveness of the communication. Finally, the advertisement of the Sport-Santé booth prior to and during these events might have been insufficient. Interestingly, one public event (*Marathon Indoor Cycling*—October 11, 2015) induced a noticeable increase (from 27 to 83 visits) in the daily number of visits to www.sport-sante.lu (see peak in October 2015 in Figure 2). This increase, however, was not statistically significant in our model. This event had a large media coverage (i.e., press, TV, radio), which had probably contributed to an increase in the number of visits to the counterfactual websites as well. Our event study also identified two initiatives (*Salon Top Sport, City Concorde*), which were followed by a significant decrease in number of visits compared to the expected number of visits of our model. This result shows the difficulty to maintain the attractiveness of a website over the time.

The use of the event study analysis to evaluate the effectiveness of communication is interesting and can help identify initiatives which are worth organizing. Nevertheless, this method should be considered complementary to other evaluation methods, both quantitative (number of visits at events, number of flyers distributed, requests for additional information, number of articles published in the media after participation in an event) and qualitative (top of mind awareness, tone of press coverage, etc.). In addition, the groups’ factsheets and the theoretical factsheets were the most viewed sections of www.sport-sante.lu just after its homepage. This reflects the interest of the visitors on the content of the website, which is actually used as a tool to inform about local physical activity offers and to explain the rationale of being physically active with a chronic disease.

The purpose of increasing the number of visits to the website if there is no increase in participation in the physical activity groups can be debated. Indeed, there still may be benefits for the patients (e.g., reading the factsheets etc.). Even if an initiative did not increase the number of visits to the website, it could still have had an impact *via* other channels such as the dissemination of the existence of the project. Nevertheless, new and unique strategies should be explored to promote physical activity among patients with NCDs. Infographics (an abbreviated term for “information graphic”) could be used more often to sensitize patients and the general population (20). Indeed, we are likely to remember up to 6.5 times more through learning from an infographic than by reading text alone (21). Infographics should therefore be used more in popular science publications and on posters and websites; they can also be disseminated easily on social networks (e.g., Facebook, Twitter, etc.). Furthermore, information booths could include nudging elements to promote Sport-Santé and its website more efficiently (e.g., a tablet displaying the website, complimentary products branded with the website address, a quiz requiring connection to the website). However, future studies are needed to evaluate the effectiveness of infographics and nudging promotions (22). Exercise “prescription” by clinicians may be the best way to promote physical activity among patients with NCDs. Unfortunately, despite the calls to action of different scientific and medical organizations, most medical doctors do not assess or prescribe physical activity as a part of routine care (23, 24). Various factors influence this behavior, which could be considered as a medical negligence (25). Indeed, the physician’s own exercise habits, lack of time, overestimation of the adverse effects of physical activity, fear of litigation, and limited knowledge have been cited as explanations (25). In Luxembourg, physical activity prescription is not systematically used, as it is not yet reimbursed by the national health fund. This important step in the promotion of physical activity for patients with NCDs should be advanced at the policy level and added to the political agenda. International and national advocacy (e.g., *via* WHO, HEPA Europe network, Exercise is Medicine^®^, Exercise Works, etc.) will contribute to reaching this target. Health advocacy, defined as the “combination of individual and social actions designed to gain political commitment, policy support, social acceptance and systems support, for a particular health goal or program” (26) is growing in Luxembourg (e.g., Sport-Santé, organizations offering physical activity for patients with NCDs, etc.). Generally, efficient use of advocacy and communication to convince more people with NCDs to be more active is required. For this particular topic, there is sufficient evidence to act. Much better use of well-planned, coherent communication strategies are needed at both national and international levels (26).

Our study is not without limitations. Usually, the control period of an event study is longer than 120 days. Our control period was relatively short (only 69 days). Immediately after the launch of the Sport-Santé website (which was announced through a press conference), the number of visits varied consistently from 1 day to another for 2½ months (from April 22, 2015 to July 5, 2015). This period was excluded from the analysis and therefore reduced the length of the control period, which ended with the first communication event. In addition, we did not track if the traffic on the website was due to new or recurrent visitors. Other limitations concern the contents of the initiatives (articles, booths), the delay between the initiative day and the website visits, and the absence of systematic website consulting after the initiatives. For instance, a pop-up, opening with each website visit, was considered in order to investigate more precisely how visitors heard about the website. Yet, we did not implement this solution because of the high probability that such pop-up windows would be an annoyance to visitors. For the e-communication (e.g., social networks), specific links provided by Google Analytics could be used to help track the origins of the visitors. The Sport-Santé promotion did not use television or radio or similar advertisements, and we do not know if these would have had a larger effect on the level of public awareness of the project. The main limitation of our study is the relationship between the increase in website visits and the increase in participation in physical activity. We assumed that, if more people were aware of the project and its offer, more people would have been likely to participate. Behavioral change (e.g., engaging in physical activity for sedentary people with NCDs) is a complex process, however, although there is a link between the website (i.e., the knowledge) and participation. According to the transtheoretical model of behavior change, comprising five stages (pre-contemplation, contemplation, preparation, action, maintenance) (27), knowledge of the offer and the benefits in engaging in a more physically active lifestyle are required to enter the contemplation phase. Obviously, other strategies, such as prescription (23) and/or motivational interviewing (28, 29), are necessary to persuade people with NCDs to advance from one stage to other, which should result in an increased participation. Further studies should be done to examine if website visits do or do not result in increased participation and whether prescription and behavioral change strategies may be added and combined (30). The results of this study should be interpreted in light of these limitations.

In conclusion, this event study showed that the impact of the most initiatives promoting the Sport-Santé project to increase the number of visits to its website was limited. Nevertheless, promoting physical activity for people with NCDs can be effective through interactive workshops and massive non-specialized press publications. The advertisement of the offer of physical activity is an important step in the complex process of lifestyle change for people with NCDs and should be accompanied by prescription or behavioral change strategies. Making every contact count, however, is essential for physical activity promotion; sustainable strategies are needed to address the problem in as many ways as possible, including through websites such as ours.

## Author Contributions

AL, JT, MV, and CH conceived and designed the study. AL, JT, MV, JP, EB, and CH performed the data collection, data entry, and analysis. CD, RS, AU, and DT supervised this research process. AL, JT, MV, JP, EB, CH, CD, RS, AU, and DT wrote, drafted, and revised the manuscript. All the authors approved the final manuscript.

## Conflict of Interest Statement

The authors declare that the research was conducted in the absence of any commercial or financial relationships that could be construed as a potential conflict of interest.

## Footnotes

^1^http://www.sport-sante.lu/index.php/en/2015-02-20-09-46-44/cardiovascular-diseases-2/association-luxembourgeoise-des-groupes-sportifs-pour-cardiaques-section-centre.

^2^http://www.sport-sante.lu/index.php/en/2015-02-20-09-46-44/cancer-2/fondation-cancer.

^3^http://www.sport-sante.lu/index.php/en/2015-02-20-09-46-44/neurologic-rare-diseases-2/parkinson-luxembourg.

^4^http://www.sport-sante.lu/index.php/en/physical-activity-and-diseases/cardiovascular-diseases/coronary-heart-disease.

^5^http://www.sport-sante.lu/index.php/en/physical-activity-and-diseases/cancer/breast-cancer.

^6^http://www.sport-sante.lu/index.php/en/physical-activity-and-diseases/neurologic-rare-diseases/parkinson-s-disease.

^7^http://www.sport-sante.lu/index.php/en/events.

^8^http://www.sport-sante.lu/index.php/en/documents-press.

^9^http://www.cancer.lu/sites/cancer/files/files/IC82_BD.pdf.
